# The Effect of Timing of Debridement and Surgical Intervention in Open Fractures on the Rate of Infection and Surgical Outcomes: A Prospective Study in a Tertiary Care Setup

**DOI:** 10.7759/cureus.37204

**Published:** 2023-04-06

**Authors:** Arpit Singh, Ankur Agarwal, Ravindra Mohan, Shailendra Singh, Prakash Tewari, Shubham Srivastava

**Affiliations:** 1 Department of Orthopedic Surgery, King George's Medical University, Lucknow, IND; 2 Department of Orthopedic Surgery, Jawaharlal Nehru Medical College, Aligarh, IND; 3 Department of Pediatric Orthopedic Surgery, King George's Medical University, Lucknow, IND

**Keywords:** gustilo-anderson classification, orthopedic emergency, compound fractures, debridement, infection rate

## Abstract

Introduction

Open fractures remain one of the true orthopedic emergencies. Despite recent advances in orthopedic surgery, the management of compound fractures is still a challenge to an orthopedic surgeon. Open fractures are a result of high-speed injuries and are associated with several complications such as infections, non-unions, or sometimes an eventual amputation. Infection is the major problem associated with open fractures due to soft tissue damage, contamination, and neurovascular compromise. Presently, management of open fractures requires early aggressive debridement followed by limb salvage by definitive reconstruction or amputation, depending upon the extent and location of the injury. Early aggressive debridement of open fractures has always been the rule. However, it has been observed that open fractures managed even after six hours of injury fare well, and there are no definite guidelines available to decide the safe period of debridement following open fractures so as to prevent infection. The "six-hour rule" is a hotly debated topic with fervent perseverance of this dogma despite a gross lack of support from the literature.

Objective

The objective of this study was to analyze the relationship between the timing of operation/debridement on infection rates in open fractures, particularly if surgery is performed after six hours.

Methods

This is a prospective study of 124 patients (R=5-75 years) presenting with open fractures to the outpatient department (OPD) and emergency section of a tertiary care hospital from January 2019 to November 2020. Patients were divided into four groups based on the time to operation/debridement: groups A, B, C, and D, with patients operated within six hours, six to 12 hours, 12-24 hours, and 24-72 hours after injury, respectively. Infection rates were obtained based on the above data. ANOVA was applied using SPSS 20 software (IBM Inc., Armonk, New York).

Results

This study concludes that the infection rate for fractures treated in less than six hours was 18.75%; in the six to 12 hours group, it was 18.50%, and in the 12-24 hours group, it was 14.28%. The infection rate increased to 38.8% if surgery was performed after 24 hours of injury. On statistical analysis, the time to debridement was not found to be a significant factor. The infection rate in Gustilo-Anderson classification compound grade I was 2.7%, grade II 9.8%, grade IIIA 45%, and grade IIIB 61%. Also, in this study, the union rate in grade I was 97.22%, grade II 96.07%, grade IIIA 85%, and grade IIIB 66.66%. Thus, the degree of wound contamination and compounding gives a prognostic indication regarding the final outcome of the compound fracture.

Conclusion

Time to debridement is not a significant factor in the management of compound fractures, and these fractures can be safely debrided up to 24 hours after injury. Gustilo and Anderson's classification provides a prognostic indicator of the outcome of a compound fracture. Infection rates and non-union rates increase with increasing grades of compound fractures.

## Introduction

Compound fractures are orthopedic emergencies, and timely intervention is the key to their management. However, in densely populated developing countries where high-velocity vehicular accidents are quite common, timely management of these fractures is still challenging. Management of compound fractures has undergone a drastic change over the last two decades. Presently, management of open fractures requires early aggressive debridement followed by limb salvage by definitive reconstruction or amputation, depending upon the extent and location of the injury [1-5]; this requires the cooperation of orthopedic, plastic, and vascular surgeons in trauma centers. The treatment protocols aimed at reducing infection and achieving optimal outcomes have evolved and include prompt resuscitation, prophylactic antibiotics, and wound irrigation and debridement [2,3,6]. Wound debridement is a cornerstone in the management of compound fractures. Traditionally, it has been maintained that wound debridement of such fractures should be carried out within six hours of injury. Based on the results of his basic research, the "six-hour rule" for debridement was developed by Friedrich, a German physician [6-13]. Debridement within this period decreases the likelihood of infection and improves the overall outcome [12,14-19]. This is seldom achievable because of delayed presentation of the patient to the hospital and other unavoidable delays after arriving at the hospital. Prompt restoration of function and union and preventing complications such as infections are the principal goals in treating compound fractures. The cost-effectiveness of the treatment is an important secondary concern while managing such injuries in a developing country. There is little literature available to give an insight into the infection rates in compound fractures operated after six hours of injury. This study was carried out to analyze the effect of timing of debridement after the injury on infection rates in compound fractures.

## Materials and methods

Inclusion and exclusion criteria

Patients of all age groups and genders who sustained injuries to the extremities and presented within 72 hours of injury were included in this study. Patients who presented after 72 hours of injury or those operated on after 72 hours due to associated comorbidities and other injuries (chest, abdominal, and head injuries) were excluded from the study. Patients were informed about the nature of the study, and written consent was taken from all patients (Institutional Ethical Committee of King George's Medical University; reference code 116th ECM IIA/P14).

Surgical methods for emergency treatment

The vitals of the patients were assessed, and initial management based on the advanced trauma life support protocol guidelines was followed. A primary survey of patients was conducted to address life-threatening conditions. A preliminary examination of the wound was carried out, and findings were documented. The wound was covered with a sterile dressing after gentle cleaning and appropriate splinting of the injured extremity. All patients were administered parenteral broad-spectrum antibiotics to cover gram-negative, gram-positive, and anaerobic bacteria, along with anti-tetanus prophylaxis [20]. In addition, the required radiological and hematological investigations were conducted. The patient was thoroughly evaluated by the anesthetist regarding the general condition and type of anesthesia.

Operative management

After the successful induction of anesthesia, skin cleansing was carried out with a povidone-iodine scrub solution while covering the wound with a saline dressing. Thorough irrigation of the wound was conducted with hydrogen peroxide and later with saline, as per Anglen recommendations (3L for grade I, 6L for grade II, 9L for grade IIIA, and 12L for grade IIIB). Debridement was carried out in an orderly fashion, beginning with the skin, progressing to the depths of the wound, and avoiding the exposed neurovascular structures. All necrotic tissue, including bone, was removed from the wound. Wound irrigation was conducted after the debridement [21-25].

Fracture fixation

Essentially, the management of compound fractures involves skeletal stabilization with correction of axial and rotational malalignment. A careful restoration of the distorted anatomy of the traumatized tissue is conducted, unkinking the vessels and thereby enhancing the perfusion to the local tissue and improving the overall circulation to the injured zone. The decision for internal/external fixation of the fracture depends upon the condition of the soft tissue and the degree of contamination. Even in the case of fractures that necessarily require internal fixation as definite management, such as intra-articular fractures, it is the soft tissue envelope that dictates the type and timing of surgery. Internal fixations that require additional soft tissue dissection cause further soft tissue trauma; thus, internal fixation in fractures with tenuous surrounding soft tissue must be delayed, thereby giving the soft tissue time to heal (e.g., pilon fractures, fractures of the tibial plateau) [25]. Fixation in injuries with heavily contaminated wounds, especially those involving the cancellous regions of bone, must be delayed. In our study, out of 130 fractures, 104 patients were managed by internal fixation, whereas 26 patients were managed by external fixation.

Wound closure and prophylactic antibiotics

The extension of the wound performed at the time of debridement was closed as long as the edges could be brought together without any tension. The types of closure of the wound were: (A) primary closure, where the wound was primarily closed over the suction drain in those cases where closure could be achieved without tension or when there was not much soft tissue or skin loss after wound debridement. This procedure is followed when the wound is not severely contaminated. This procedure converts an open fracture into a closed one. (B) Delayed primary closure: when the wound is left open, it is repeatedly debrided at 24-hour intervals, and if the wound is found healthy with no signs of infection, it is closed after three to five days of injury. (C) Secondary closure: in a few cases, the wound was left open and daily dressing was carried out. Debridement was conducted whenever required, and the wound was allowed to granulate. The wound was secondarily closed 10-15 days after the injury. And (D) wound coverage: skin defects were covered using split-thickness skin grafts. Fascio-cutaneous and myocutaneous flaps were used to cover bone and tendons. In the postoperative period, all patients were prescribed broad-spectrum antibiotics to cover gram-positive, gram-negative, and anaerobic bacteria. The parenteral route was used for the first seven days, and oral antibiotics were given after wound inspection. The spectrum of infection included any purulent discharge from the wound, the presence of an abscess in the wound or stitch line, wound dehiscence, infected foreign body including metalware, and osteomyelitis.

Postoperative treatment and follow‐up postoperative treatment

At stitch removal, wounds were assessed for any gaping/discharge. If any discharge was present, it was sent for pus culture and sensitivity. These findings were recorded in the proforma, and the patients were followed in an outpatient department (OPD) at regular intervals of four to six weeks. Their progress was assessed in terms of presenting complaints, wound condition, condition of pin tracts in case of external fixation, range of motion, and clinical/radiological signs of infection.

Debridement time grouping and time to surgery

The time from injury to debridement was recorded for each case. Patients were divided into four groups based on the time until operation/debridement. In group A, patients were operated on within six hours after injury; in group B, patients were operated on six to 12 hours after injury; in group C, patients were operated on 12-24 hours after injury; and in group D, patients were operated on 24-72 hours after injury. Patients who were operated on after 72 hours of injury were excluded from this study.

Gustilo‐Anderson classification grades

Grade I is an open fracture with minimal contamination, comminution, and soft tissue damage, wound <1 cm in length. Grade II is an open fracture with moderate contamination, comminution, and soft tissue damage, wound >1 cm but less than 10 cm in length. Grade III in an open fracture with extensive contamination, comminution, and soft tissue damage, wound >10 cm in length. Grade IIIA is a fracture with crushed tissue and contamination but usually with adequate soft tissue coverage. Grade IIIB is a fracture with extensive soft-tissue loss and periosteal stripping, and bone damage that will need further soft-tissue coverage procedure. Grade IIIC is a fracture associated with an arterial injury-requiring repair, irrespective of the degree of soft-tissue injury (Table 1).

**Table 1:** Gustilo-Anderson classification

Grade	Description
Grade I	Open fracture, minimal contamination, comminution, and soft tissue damage, wound <1 cm in length
Grade II	Open fracture, moderate contamination, comminution, and soft tissue damage, wound > 1 cm but less than 10 cm in length
Grade III	Open fracture, extensive contamination, comminution, and soft tissue damage, wound > 10 cm in length
III A	Type III fracture with crushed tissue and contamination but usually with adequate soft tissue coverage
III B	Type III fracture with extensive soft-tissue loss and periosteal stripping and bone damage. Will need further soft-tissue coverage procedure
III C	Type III fracture associated with an arterial injury-requiring repair, irrespective of degree of soft-tissue injury

Statistical analysis

All statistical analyses were performed using IBM SPSS statistics version 20.0 (IBM Inc., Armonk, New York), and ANOVA was applied. For quantitative variables, the data between the groups were expressed as mean ± SD and compared by a t‐test for statistical analysis. A value of p<0.05 indicated a statistically significant difference.

## Results

The following observations were made in this study of 130 patients presenting with compound fractures to the emergency and OPD of a tertiary care hospital. Six patients were lost to follow-up. The final results are based on 124 patients with 130 fractures presenting from January 2014 to November 2015; these were prospectively followed and analyzed. The basic information of the patients is shown in the tables below. In our study, according to Gustilo and Anderson's classification system (Table 1), a maximum number of patients sustained compound grade II fractures (39%), followed by compound grade III fractures (33%). Furthermore, 15.38% of the patients had grade IIIA fractures, 13.84% had grade IIIB fractures, 3.84% had grade IIIC fractures, and 27% had grade I fractures (Table 2).

**Table 2:** Number of fractures and Gustilo-Anderson classification grades of compounding

Grade of compounding	Number of fractures	Percentage (%)
Grade I	36	27.69
Grade II	51	39.23
Grade III-A	20	15.38
Grade III-B	18	13.84
Grade III-C	5	3.84
Total	130	100.00

A total of 70% of the patients in our study presented to the hospital within six hours of injury, while only eight out of 124 (6.45%) patients presented after 24 hours (Table 3).

**Table 3:** Time between injury and presentation of patients

Time	Number of patients	Percentage (%)
0-3 hours	40	32.25
3-6 hours	48	38.70
6-12 hours	22	18.46
12-24 hours	6	4.8
After 24 hours	8	6.45
Total	124	100.00

Most of the patients were debrided and operated on or within six to 12 hours after injury (41.53 %), followed by 12-24 hours (32.30%); only 12% of patients could be operated on within six hours of injury (Table 4).

**Table 4:** Debridement time grouping and time to surgery

Time	Number of fractures	Percentage (%)
0-6 hours (group A)	16	12.30
6-12 hours (group B)	54	41.53
12-24 hours (group C)	42	32.30
24-72 hours (group D)	18	13.84
Total	130	100.00

This study found that the infection rate for fractures treated in less than six hours was 18.75%, while in the six to 12 hours group, the rate was 18.50%, and in the 12-24 hours group, 14.28%. The infection rate increased to 38.8% if surgery was performed after 24 hours of injury. On statistical analysis, the time to debridement was not found to be a significant factor (Table 5). 

**Table 5:** Infection rate based on the timing of operation

Groups	Total number of fractures	Number of fractures with infection	Infection rate (%)
Group A (0-6 hours)	16	3	18.75
Group B (6-12 hours)	54	10	18.50
Group C (12-24 hours)	42	6	14.28
Group D (24-72 hours)	18	7	38.80
Total	130	26	20.00

The infection rate in compound grade I was 2.7%, in grade II 9.8%, in grade IIIA 45%, and in grade IIIB 61%. The union rate in grade I was 97.22%, in grade II 96.07%, in grade IIIA 85%, and in grade IIIB 66.66% (Table 6). 

**Table 6:** Infection rate based on the grade of compounding

Grade	Number of fractures	Number of fractures with infection	Infection rate (%)
Grade I	36	1	2.70
Grade II	51	5	9.80
Grade III-A	20	9	45.00
Grade III-B	18	11	61.00

A total of 101 fractures united over the period of the study. There were 12 cases of non-unions and five delayed unions. Ten patients still had uniting fractures at the time of the conclusion of the study. Four out of five compound grade IIIC fractures underwent amputation.

## Discussion

With advancements in modern technology and people's fast-paced lifestyle, the incidence of compound fractures is on the rise. In this study, more than half of the patients (63.7%) sustained compound fractures in road traffic accidents. The most common group of the population involved in compound fractures was young adults (age group 21-30 years). Out of 124 patients included in this study, the majority were males (84%). This can be explained by men being more involved in outdoor activities on our subcontinent and hence having relatively more exposure to outdoor accidents compared to the female population.

In our study, most of the compound fractures belonged to grade II (39%), followed by grade III (33%) (Table 1), and most of the patients (70%) presented to our hospital within six hours of injury (Table 2). However, preparing the patient for surgery caused inadvertent time losses. Most of the patients could be operated on between six to 12 hours after injury (41.53%), followed by 12-24 hours (32.30%) (Table 3). Only 12% of patients could be operated on within six hours of injury. Numerous causes contributed to delays in performing surgery, such as resuscitation and preparation of the patient, preparation of the emergency operating theater (OT), availability and sterilization of implant, availability of the anesthesia team, and financial problems of the patient.

In our study, it was found that the infection rate for fractures operated within six hours was 18.75%. In the six to 12 hours group, the infection rate was 18.50%, and in the 12-24 hours group 14.28%. The infection rate increased to 38.8% when surgery was performed after 24 hours of injury (Table 4). Thus, this study concludes that infection rates in compound fractures were not affected by the time of operation, even if surgery was delayed up to 24 hours [20-24]. On bi- and multivariable analysis, the time from injury to debridement was not found to be a significant prognostic factor in our study [24-27]. Furthermore, no control group of patients presenting with compound fractures underwent a deliberate delayed debridement. However, these results do not indicate that early debridement of compound fractures resulted in a lower rate of infection; knowing if the time from injury to debridement affects infection rates would require a randomized controlled trial (RCT). To ensure that the various confounding factors related to patients (e.g., diabetes mellitus, smoking), fractures (e.g., grade and severity of the injury, site of injury), and the treating surgeons (e.g., experience, choice of fixation method) does not affect the result of the study, a large number of cases would be required, which is a difficult task. Moreover, delaying a patient's treatment for the purpose of a trial would be unethical. Thus, the dictum of six hours as the cut-off limit for emergent treatment seems to be based more on historical precedence than scientific evidence. It can be concluded from this study that, in some cases, it may not be in the patient's best interest to operate within six hours. This may lead to complex cases being performed at night hours by relatively inexperienced surgeons, anesthetists, and theatre staff with tired minds and bodies, thus resulting in less favorable outcomes. A limitation of our study is that it is a single-center prospective study. All surgeries were performed by multiple surgeons in the hospital and thus had differences in surgical hands. Therefore, we recommend the research results to be further confirmed by multi-center large‐sample studies.

## Conclusions

This study demonstrates that the time to debridement is not a significant factor in the management of compound fractures and these fractures can be safely debrided up to 24 hours of injury. The Gustilo-Anderson classification provides a prognostic indicator of the outcome of a compound fracture. Infection rates and non-union rates increase with increasing grades of compound fractures. Further, early soft tissue cover is recommended, as it decreases the chances of infection, the cost of treatment, and the length of hospital stay.
